# Baduanjin exercise modulates the hippocampal subregion structure in community-dwelling older adults with cognitive frailty

**DOI:** 10.3389/fnagi.2022.956273

**Published:** 2022-12-19

**Authors:** Mingyue Wan, Rui Xia, Huiying Lin, Yu Ye, Pingting Qiu, Guohua Zheng

**Affiliations:** ^1^School of Rehabilitation Sciences, Southern Medical University, Guangzhou, China; ^2^College of Nursing and Health Management, Shanghai University of Medicine and Health Sciences, Shanghai, China; ^3^College of Rehabilitation Medicine, Fujian University of Traditional Chinese Medicine, Fuzhou, China; ^4^Department of Rehabilitation, Shenzhen Bao ‘an District People’s Hospital, Shenzhen, China

**Keywords:** Baduanjin, cognitive frailty, hippocampal subregion, structural plasticity, MRI

## Abstract

**Background:**

Regular Baduanjin exercise intervention was proven to be beneficial in improving the cognitive ability and physical performance of older adults with different health conditions but was unclear to influence the structural plasticity of the hippocampus. This study aimed to explore the modulation of hippocampal subregions as a mechanism by which Baduanjin exercise improves cognitive frailty in older adults.

**Methods:**

A total of 102 community-dwelling older adults with cognitive frailty were recruited and randomly allocated to the Baduanjin exercise training group and usual physical activity control group. The participants in the Baduanjin exercise training group participated in a 24-week Baduanjin exercise intervention program with an exercise frequency of 60 min per day, 3 days per week. Cognitive ability and physical frailty were assessed, and MRI scans were performed on all participants at baseline and after 24 weeks of intervention. The structural MRI data were processed with MRIConvert (version 2.0 Rev. 235) and FreeSurfer (version 6.0.0) software. Data analyses were performed using the independent sample *t* tests/Mann–Whitney *U* tests with the Bonferroni correction, mixed linear model, correlation, or mediation analysis by the SPSS 24.0 software (IBM Corp, Armonk, NY, United States).

**Results:**

After 24 weeks of intervention, a statistically significant increase was found for the Montreal Cognitive Assessment (MoCA) scores (*p* = 0.002) with a large effect size (Cohen’s *d* = 0.94) and the significant interaction effect (*P*_goup × time_ < 0.05), Memory Quotient (MQ) scores (*p* = 0.019) with a medium effect size (Cohen’s *d* = 0.688) and the significant interaction effect (*P*_goup × time_ < 0.05), and other parameters of WMS-RC test including pictures (*p* = 0.042), recognition (*p* = 0.017), and association (*p* = 0.045) test with a medium effect size (Cohens’ *d* = 0.592, 0.703, and 0.581) for the Baduanjin training group, while significant decrease for the Edmonton Frailty Scale (EFS) score (*p* = 0.022), with a medium effect size (Cohen’s *d* = −0.659) and the significant interaction effect (*P*_goup × time_ < 0.05) for the Baduanjin training group. The differences in the left parasubiculum, Hippocampal Amygdala Transition Area (HATA), right Cornu Ammonis Subfield 1 (CA1) and presubiculum volumes from baseline to 24 weeks after intervention in the Baduanjin training group were significantly greater than those in the control group (*p* < 0.05/12). Further analysis showed that the changes in right CA1 volume were positively correlated with the changes in MoCA and MQ scores (*r* = 0.510, *p* = 0.015; *r* = 0.484, *p* = 0.022;), the changes in right presubiculum and left parasubiculum volumes were positively correlated with the changes in MQ (*r* = 0.435, *p* = 0.043) and picture test scores (*r* = 0.509, *p* = 0.016), respectively, and the changes in left parasubiculum and HATA volumes were negatively correlated with the changes in EFS scores (*r* = −0.534, *p* = 0.011; *r* = −0.575, *p* = 0.005) in the Baduanjin training group, even after adjusting for age, sex, years of education and marital status; furthermore, the volume changes in left parasubiculum and left HATA significantly mediated the Baduanjin exercise training-induced decrease in the EFS scores (*β* = 0.376, 95% CI 0.024 ~ 0.947; *β* = 0.484, 95% CI 0.091 ~ 0.995); the changes of left parasubiculum and right CA1 significantly mediated the Baduanjin exercise training-induced increase in the picture and MO scores (*β* = −0.83, 95% CI-1.95 ~ −0.002; *β* = −2.44, 95% CI-5.99 ~ −0.32).

**Conclusion:**

A 24-week Baduanjin exercise intervention effectively improved cognitive ability and reduced physical frailty in community-dwelling older adults with cognitive frailty, and the mechanism might be associated with modulating the structural plasticity of the hippocampal subregion.

## Introduction

Cognitive frailty (*CF*) refers to a state characterized by the presence of both physical frailty and cognitive impairment in nondemented older adults (Buchman and Bennett, 2013), and is associated with increased risk of dysfunction, deterioration of quality of life, hospitalization, mortality, dementia and neurocognitive impairment (Panza et al., 2018). Although the underlying mechanism of *CF* remains unclear, a direct link has been proposed between brain pathology and cognitive frailty. An increasing number of studies have demonstrated the association of structural brain changes with the pathogenesis of cognitive frailty (Sugimoto et al., 2022). Current evidences showed the hippocampus or its subregion played an important role in memory consolidation as well as in energy intake, behaviors and mood regulation, therefore associated with cognitive and body function (Davidson et al., 2007; Bettio et al., 2017). For example, a study reported that hippocampal subfields including cornu ammonis 1 (CA1), CA2/3 and CA4 were significantly covaried with grey matter volume in older adults with cognitive impairment compared to the normal controls (Wang et al., 2018). Another study also found that the larger left parahippocampal gyrus and right hippocampus volumes were associated with the higher physical activity ability (Domingos et al., 2021). In our previous studies, we found a significant decrease in the volume of subcortical nuclei (composing of hippocampus, thalamus, caudate, putamen, pallor and amygdala) in the *CF* older adults (Wan et al., 2020). Then we further found a significant atrophy of six hippocampal subregions in *CF* older adults, including the left presubiculum, left parasubiculum, left molecular layer of the hippocampus proper (molecular layer of the HP), left HATA, right presubiculum, and right cornu ammonis subfield 1 (CA1; Wan et al., 2020). Therefore, changes in hippocampal subregion structure might play an important role in the pathogenesis of cognitive frailty.

Physical activity or regular exercise has been deemed an effective intervention for reducing physical frailty and increasing cognitive ability (Bherer et al., 2013; Angulo et al., 2020), and therefore should be a promising approach for improving cognitive frailty (Liu et al., 2018). For example, the community-based exercise program (i.e., a kind of exercise mode for community-dwelling older adults including multi-component exercise training with a low-moderate intensity) was effective in improving quality of life and physical frailty of community-dwelling older adults (Julien, 2021), while the aerobic, resistance or multicomponent exercise was proved to increase the cognitive function in older adults with or without cognitive impairment (Lee, 2020; Venegas-Sanabria et al., 2022). Moreover, converging evidences also suggest that the cognitive and physical performance improvement of exercise intervention may be brought about by enhanced structural network integrity of the human brain (Pani et al., 2021; Won et al., 2021; Huang et al., 2022). As one of the most popular traditional mind–body exercises in China, Baduanjin exercise consists of eight movements with low-medium intensity and is characterized by symmetrical body postures and movements, breathing control, a meditative state of mind, and mental focus (Koh, 1982). Different from other types of physical exercise, Baduanjin emphasizes the mindfulness and breathing integration practice by cultivating *qi* (a vital energy based on traditional Chinese medicine) to improve physical, mental and cognitive health, and is recommended for older adults in China (Zheng et al., 2016; Tao et al., 2017; Zou et al., 2017). Our previous study found that regular Baduanjin intervention could increase the connection of resting function between the bilateral hippocampus and prefrontal lobe, and effectively prevent the decline of memory in the process of aging (Tao et al., 2016). This study aimed to explore the mechanism related to the central nervous system by which Baduanjin improves cognitive frailty in older adults from the perspective of the hippocampal subregions.

## Materials and methods

### Study design

This study was designed as a randomized controlled trial and has been registered in the China clinical trial registration center with the registration number ChiCTR1800020341.^1^ This trial was approved by the Medical Ethics Committee of the Second People’s Hospital of Fujian Province (approval number 2018-KL015). More details about the study design were described in previous published protocol (Xia et al., 2020).

### Participants

Participants were recruited from three communities (Niushan, Wenquan and Wufeng communities) in Fuzhou city, China. Eligible participants met the following criteria: cognitive frailty with mild cognitive impairment [Fuzhou Version Montreal Cognitive Assessment (MoCA) ≤ 26 points]; physical frailty (EFS ≥ 5 points), absence of dementia (Global Deterioration Scale (GDS) level of II or III); aged 60 years or older; no regular exercise in the past half a year; and signed informed consent. Those with a history of mental illness (such as personality disorder, schizophrenia, etc.), depression (Beck Depression Scale > 10 points), severe aphasia and visual impairment, severe organ failure, cerebral hemorrhage, cerebral infarction, history of coronary heart disease, musculoskeletal system diseases and other sports contraindications, hypertension and uncontrollable blood pressure (systolic blood pressure greater than 160 mmHg or diastolic blood pressure greater than 100 mmHg); metal implants (such as pacemakers, fixed metal dentures, etc.) and other conditions that were not suitable for MRI scanning, history of alcohol or drug abuse, or participation in other clinical trials were excluded.

### Intervention

A total of 102 eligible participants were enrolled, and were randomly allocated into the Baduanjin training group or the control group with equal ratio. Participants in the Baduanjin group participated in Baduanjin training for 24 weeks, three times a week. Each training session lasted for 60 min, including 15 min of warm-up, 40 min of Baduanjin training, and 5 min of cool down. Health education on nutrition and diet related knowledge for the elderly was conducted every 4 weeks (at least 30 min per session). Baduanjin training was implemented in the Niushan, Wenquan and Wufeng communities with 15–20 participants from each community. Professional coaches of Fujian University of Traditional Chinese Medicine were responsible for leading Baduanjin practice. Participants in the control group did not receive any specific exercise training except for the same health education on nutrition and diet as the Baduanjin exercise training group. They were asked to maintain their original activity habits.

### Cognitive and physical frailty assessment

Global cognitive function was assessed by using the Fuzhou version of the Montreal Cognitive Assessment (MoCA), which includes several cognitive domain dimensions (Fang et al., 2017) such as visuospatial, executive function, naming, memory, attention, language, abstraction, and orientation. The total score is 30, and a higher score indicates better global cognitive function. Memory was assessed using the Wechsler Memory Scale-Revised, Chinese version (WMS-RC). The MQ was the total score of WMS-RC which calculated according to age (Elwood, 1991). Higher MQ score denoted better memory. The WMS-RC was a set of memory tests that could detect impairment of long-term, short-term, and transient memory. Long-term memory includes counting 1–100, counting 100–1, accumulate subtests; short-term memory includes recognition, pictures, regenerate, association, touch and understand subtests; transient memory includes recite numbers subtests. Physical frailty was assessed by using the Chinese version of the Edmonton frail scale (EFS) with a total of 17 points, and high scores denoted a serious degree of frailty (Rolfson et al., 2006). All measures were conducted by blinded assessors at baseline and after intervention.

### Basic information acquisition

Basic information included demographic characteristics such as age, gender, marital status, years of education were collected, while cognitive deterioration and depressive symptoms were assessed by the recruiters using the self-designed questionnaire, the Global Deterioration Scale and Beck Depression Scale, respectively.

### MRI data acquisition

All participants underwent structural MRI at baseline and after intervention using a Siemens Prisma 3.0 T MRI system (Siemens Medical System, Erlangen, Germany) at the Rehabilitation Hospital Affiliated with FJTCM. The parameters of the structural MRI were as follows: repetition time (TR) = 2,300 ms, echo time (TE) = 2.27 ms, flip angle = 8°, slice thickness = 1.0 mm, field of view (FOV) = 250 × 250 mm, matrix = 256× 256, voxel size = 0.98 × 0.98 × 1 mm^3^, and number of slices = 160.

### Image processing

MRIConvert (version 2.0 Rev. 235) and FreeSurfer (version 6.0.0) software were used to preprocess the structural MRI data. Image processing was divided into the following steps: (1) image format conversion: MRIConvert software was used to convert the structure MRI data from the DICOM to the NIFTI format; (2) image quality inspection: each subject’s image was checked for artifacts, lesions and other abnormalities and unqualified images were excluded; (3) direction adjustment: the image direction for each subject was adjusted consistently; (4) image segmentation: gray matter, white matter, subcortical nucleus, surface of white matter and cerebrospinal fluid were segmented by using the consistent segmentation method; (5) obtaining of deformation relation: obtaining curvature deformation relation from individual structure image to standard space; (6) spatial standardization: the individual level indicators are registered into the standard space by using the upper generated deformation relationship; and (7) smoothing: differences between individuals were reduced after standardization and the signal-to-noise ratio was improved.

The volume of the hippocampus in the subcortical nucleus segmentation file for each subject was extracted by using FreeSurfer 6.0.0 software, and the hippocampus and amygdala were segmented at the same time. The joint segmentation of the two areas ensured that the structures would not overlap or have a gap between them, making the results more accurate (Saygin et al., 2017). According to the official website for FreeSurfer,^2^ each subject’s hippocampus was divided into 19 regions according to the segmentation template of the hippocampus and then combined into the following areas: hippocampal tail, subiculum, cornu ammonis 1 (CA1), hippocampal fissure, presubiculum, parasubiculum, molecular layer of the HP, granule cell layer and molecular layer of the dentate gyrus (GC-ML-DG), CA2/3, CA4, hippocampal fimbria, and hippocampal amygdala transition area (HATA; Iglesias et al., 2015). CA2 was always included in CA3.

### Data analysis

The data were analyzed by using SPSS 24.0 software (IBM Corp, Armonk, NY, United States), and *p* < 0.05 was considered significant. Independent sample t tests or Mann–Whitney U tests were used to compare the quantitative data from the two groups; Chi square tests were used to compare the categorical data of the two groups. Between-group effect size for cognitive and physical frailty outcomes were calculated using Cohen’s d, in which effect sizes of 0.2, 0.5, and 0.8 were considered small, medium, and large effects, respectively. The mixed linear model with the fixed effect was used to analyze the interaction effect of group by time for the cognitive and physical frailty variables between two groups. To examine the robustness of results on Baduanjin exercise for cognitive frailty outcomes, we conducted sensitivity analysis by including data from participants who were lost follow-up, and the missing data were filled in using the multiple imputation method.

Bonferroni correction was used in the analysis of hippocampal subregion volume, and *p* < 0.05/12 was considered significance. To explore whether Baduanjin could improve cognitive frailty by changing the plasticity of the hippocampal subregions, we analyzed the correlation between the changes in hippocampal subregion volume and the changes in cognitive and physical frailty, with sex, age, marital status, and years of education as covariates. Moreover, we also analyzed the mediation effect of changes of hippocampal subregions in the relationship between intervention and cognitive frailty measurement by using the SPSS-PROCESS v3.5 software.

## Results

**Figure 1:**
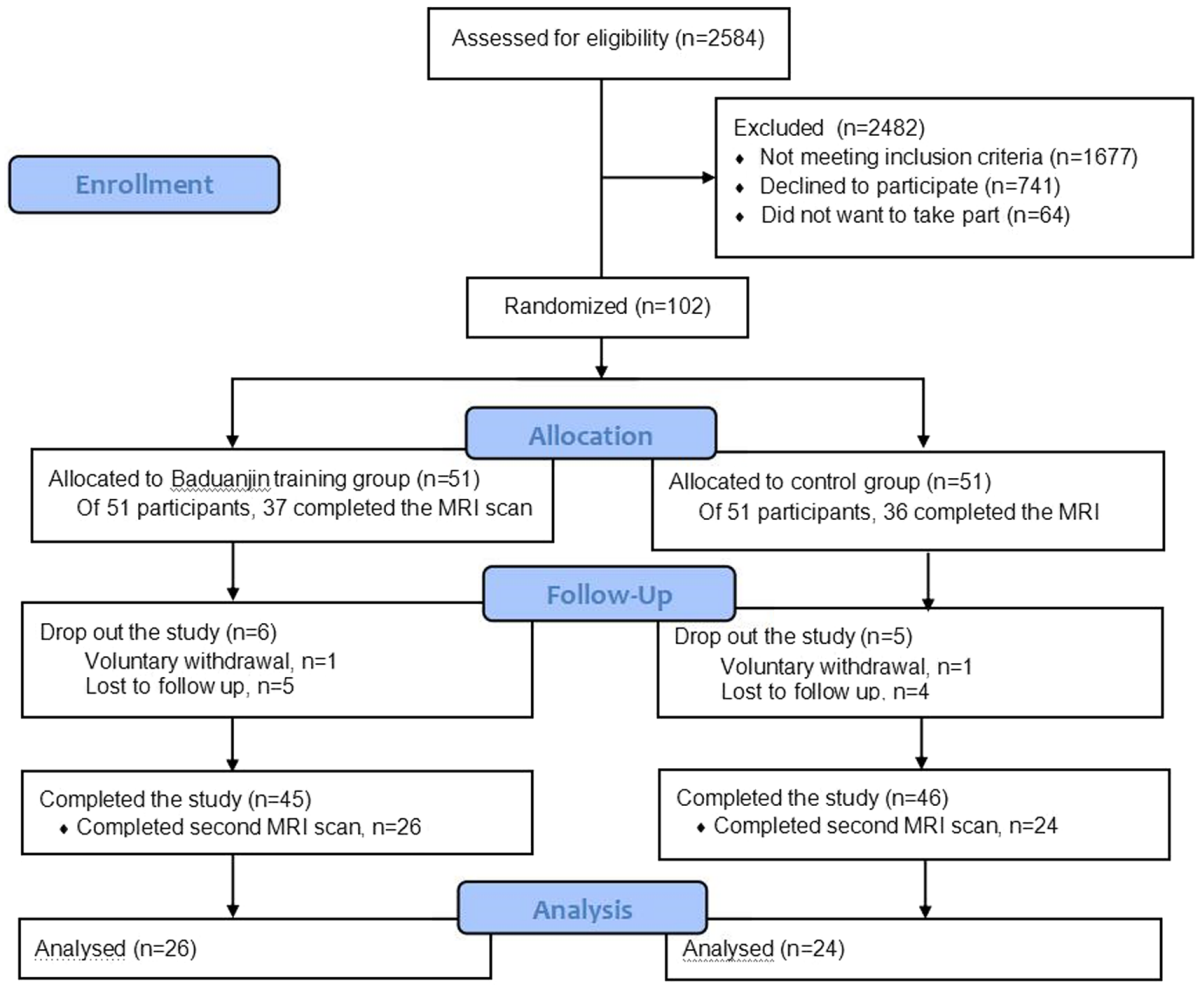
Research flow diagram.

Figure 1 shows the flow diagram of participants recruitment, randomization and follow up. A total of 102 eligible participants performed the baseline characteristics assessment and were randomly allocated into the groups. Of 102 participants, 73 were willing to perform MRI scans (37 in the Baduanjin group and 36 in the control group), and 50 of them completed the post-intervention assessment and the second MRI scans (26 in the Baduanjin group and 24 in the control group). Finally, 50 participants who completed two MRI scan were included in the analysis. For the Baduanjin exercise training group, some participants did not complete all training plans due to the limitation of bad weather and personal reasons. Even so the adherence rate was still up to 81.3%. The comparison on baseline characteristics of participants who completed two MRI scan between two groups are presented in Table 1. No significant differences were observed in basic demographic data between the two groups, including age, sex, marital status, body mass index (BMI), years of education, global deterioration scale level and Beck depression scale score.

**Table 1:** Comparison of baseline characteristics between two groups [ratio/ $\bar{X}\pm S/M$ (*P*_25_ ~ *P*_75_)].

Characteristics	BDJ (*n* = 26)	CON (*n* = 24)	*t*/*Z*/*χ*^2^	*P*
Age (year)	67.31 ± 5.58	64.71 ± 5.07	−1.843	0.065
Gender (male/female) (n)	9/17	12/12	1.213	0.271
marital status(married/widowed) (n)	25/1	24/0	–	1
BMI (kg/m^2^)	23.47 ± 2.28	24.45 ± 3.28	−1.230	0.225
Average years of education (year)	11.58 ± 2.79	10.25 ± 2.92	−1.609	0.108
Beck Depression Scale Index^*^	3.5 (1.75 ~ 5)	3.5 (3 ~ 5)	0.426	0.670
Global deterioration scale(II/III)(n)	17/9	19/5	1.176	0.278

BDJ, Baduanjin exercise group; CON, control group. BMI, body mass index. ^*^Data were performed by Mann–Whitney *U* tests.

To observe the variability on baseline characteristics between 50 participants who completed two MRI scan and 73 participants who were willing to perform MRI scan and all 102 participants, we conducted the comparison on baseline characteristics between two groups among three data sets, and those results were substantively unchanged in three data sets. The comparison on baseline characteristics between two groups among three data sets are presented in Supplementary Table 1.

### Changes in cognitive ability and physical frailty

Comparison (according to the PP analysis model) on changes of cognitive ability and physical frailty from 24-week intervention to baseline between two groups who completed two MRI scan are presented in Table 2. There was no significant difference between the two groups in terms of MoCA, WMS-RC test (both total and subtest scores) and EFS scores at baseline. After 24 weeks of the Baduanjin intervention, the independent sample *t* test or Mann–Whitney *U* test showed: the average scores of MoCA and MQ test in the Baduanjin exercise group were significantly higher than them in the control group with a medium to large effect size (*p* = 0.002 and 0.019; Cohen’s *d* = 0.94 and 0.688), and the EFS scores in Baduanjin exercise group were significantly lower than that in the control group with a medium effect size (*p* = 0.022; Cohen’s *d* = −0.659); For parameters of WMS-RC test, the scores of pictures, recognition, and association test in the Baduanjin exercise group were significantly higher than them in the control groups with medium effect sizes (*p* = 0.042, 0.017, and 0.045; Cohens’ *d* = 0.592, 0.703, and 0.581); no significant difference between groups were found in other parameters such as counting 100–1, counting 1–100, accumulate, regenerate, touch, understand, and recite number. The linear mixed model analysis showed the significant interaction effect (group × time) in MoCA, EFS scores, MQ, and counting 1–100 scores, indicating that Baduanjin exercise intervention has a differential treatment effect compared with the control group.

**Table 2:** Comparison of cognitive ability and physical frailty between groups at baseline and after 24 weeks intervention.

Variables	Groups	N	Baseline		After 24 weeks intervention			Group × time interaction *P*
			$\bar{X}\pm S/M$ (*P*_25_ ~ *P*_75_)	*P*	$\bar{X}\pm S/M$ (*P*_25_ ~ *P*_75_)	*P*	Cohen’ d	
MoCA (scores)	BDJ	26	22.50 ± 2.40	0.799	25.31 ± 2.41	**0.002**	0.940	**0.004**
	CON	24	21.88 ± 3.60		22.58 ± 3.35			
WMS-RC test (scores)								
MQ (scores)	BDJ	26	92.69 ± 13.02	0.270	102.92 ± 11.77	**0.019**	0.688	**0.026**
	CON	24	88.25 ± 15.11		93.71 ± 14.96			
Counting 1-100 (scores)	BDJ	26	7.81 ± 2.64	0.582	9.27 ± 1.87	0.235	0.349	0.015
	CON	24	8.25 ± 15.11		8.33 ± 3.34			
Counting 100-1 (scores)	BDJ	26	9.50 (6.50 ~ 11.25)	0.837	10.00 (7.00 ~ 12.00)	0.630	−0.008	0.565
	CON	24	10.00 (8.00 ~ 11.00)		11.00 (8.25 ~ 12.00)			
Accumulate (scores)	BDJ	26	10.00 (8.00 ~ 11.00)	0.896	10.00 (8.00 ~ 11.00)	1.000	0.000	0.855
	CON	24	8.25 (10.00 ~ 10.75)		9.00 (10.00 ~ 11.00)			
Pictures (scores)	BDJ	26	8.00 ± 2.67	0.955	9.81 ± 2.21	**0.042**	0.592	0.160
	CON	24	7.96 ± 2.56		8.50 ± 2.21			
Recognition (scores)	BDJ	26	8.88 ± 2.86	0.159	9.00 ± 2.84	**0.017**	0.703	0.500
	CON	24	7.58 ± 3.56		6.96 ± 2.97			
Regenerate (scores)	BDJ	26	7.00 ± 2.70	0.408	7.62 ± 2.58	0.590	0.154	0.823
	CON	24	6.38 ± 2.58		7.17 ± 3.25			
Association (scores)	BDJ	26	4.54 ± 3.43	0.205	6.23 ± 3.50	**0.045**	0.581	0.369
	CON	24	3.33 ± 3.19		4.25 ± 3.30			
Touch (scores)	BDJ	26	7.00 (6.00 ~ 7.25)	0.289	8.00 (7.00 ~ 8.00)	0.281	0.170	0.764
	CON	24	6.00 (6.00 ~ 8.00)		7.00 (6.00 ~ 8.00)			
Understand (scores)	BDJ	26	6.23 ± 1.99	0.359	7.27 ± 2.97	0.263	0.195	0.898
	CON	24	5.63 ± 2.62		6.75 ± 2.29			
Recite numbers (scores)	BDJ	26	8.27 ± 3.24	0.529	8.88 ± 3.41	0.495	0.155	0.766
	CON	24	7.71 ± 2.99		8.08 ± 2.04			
EFS (scores)	BDJ	26	5.46 ± 0.65	0.469	3.38 ± 1.33	**0.022**	−0.659	**0.016**
	CON	24	5.33 ± 0.57		4.33 ± 1.55			

BDJ, Baduanjin exercise group; CON, control group; MoCA, Montreal Cognitive Assessment; WMS-RC test, Wechsler Memory Scale-Revised, Chinese version; MQ, Memory Quotient; EFS, Edmonton Frailty Scale. Bold type *p* value represented significant.

To examine the robustness of effect on Baduanjin exercise for cognitive frailty outcomes, we conducted sensitivity analysis by including data from participants who were lost follow-up (ITT analysis model), and the results are presented in Table 3. Two analysis models yielded equivalent significant findings for the MoCA, EFS, and MQ, pictures and association of WMS-RC test though the Cohen’s *d* values of MoCA and MQ in the ITT model were a little lower than them in the PP model. Recognition parameter of WMS-RC test between two comparison groups was significant (*p* = 0.017) in the PP model but not in the ITT model (*p* = 0.059).

**Table 3:** Comparison of cognitive ability and physical frailty between groups at baseline and after 24 weeks intervention.

Variables	Groups	N	Baseline		After 24 weeks intervention			Group × time interaction *P*
			$\bar{X}\pm S$	*P*	$\bar{X}\pm S$	*P*	Cohen’ d	
MoCA (scores)	BDJ	37	22.59 ± 2.34	0.216	24.97 ± 2.57	**0.001**	0.793	**0.002**
	CON	36	21.67 ± 3.85		21.97 ± 4.72			
WMS-RC test (scores)								
MQ (scores)	BDJ	37	91.73 ± 13.06	0.155	100.97 ± 12.01	**0.025**	0.535	0.270
	CON	36	86.67 ± 16.86		93.56 ± 15.54			
Counting 1-100 (scores)	BDJ	37	7.76 ± 2.59	0.633	8.81 ± 2.42	0.506	0.157	0.151
	CON	36	8.08 ± 3.20		8.33 ± 3.58			
Counting 100-1 (scores)	BDJ	37	8.57 ± 3.44	0.692	9.46 ± 3.01	0.827	−0.051	0.385
	CON	36	8.22 ± 3.96		9.61 ± 2.88			
Accumulate (scores)	BDJ	37	9.03 ± 2.79	0.379	10.30 ± 2.25	0.174	0.321	0.946
	CON	36	8.33 ± 3.83		9.56 ± 2.37			
Pictures (scores)	BDJ	37	8.24 ± 2.58	0.419	9.70 ± 2.13	**0.025**	0.536	0.355
	CON	36	7.75 ± 2.61		8.56 ± 2.14			
Recognition (scores)	BDJ	37	8.70 ± 3.20	0.279	8.70 ± 3.02	0.059	0.449	0.598
	CON	36	7.86 ± 3.38		7.39 ± 2.83			
Regenerate (scores)	BDJ	37	7.05 ± 2.53	0.206	7.62 ± 2.41	0.783	0.065	0.366
	CON	36	6.25 ± 2.85		7.44 ± 3.05			
Association (scores)	BDJ	37	4.16 ± 3.17	0.118	6.14 ± 3.42	**0.011**	0.615	0.201
	CON	36	3.03 ± 2.94		4.06 ± 3.35			
Touch (scores)	BDJ	37	6.70 ± 2.05	0.421	7.35 ± 0.92	0.238	0.279	0.785
	CON	36	6.33 ± 1.84		6.83 ± 2.48			
Understand (scores)	BDJ	37	6.08 ± 1.99	0.312	7.22 ± 2.83	0.428	0.187	0.910
	CON	36	5.56 ± 2.41		6.75 ± 2.10			
Recite numbers (scores)	BDJ	37	8.38 ± 3.24	0.291	9.03 ± 3.06	0.112	0.376	0.759
	CON	36	7.56 ± 3.36		8.00 ± 2.34			
EFS (scores)	BDJ	37	5.38 ± 0.59	0.619	3.45 ± 1.43	**0.006**	−0.669	**0.006**
	CON	36	5.47 ± 0.97		4.50 ± 1.70			

BDJ, Baduanjin exercise group; CON, control group; MoCA, Montreal Cognitive Assessment; WMS-RC test, Wechsler Memory Scale-Revised, Chinese version; MQ, Memory Quotient; EFS, Edmonton Frailty Scale. Bold type *P* value represented significant.

### Changes in hippocampal subregions and the correlation with changes in cognitive or physical function

**Figure 2:**
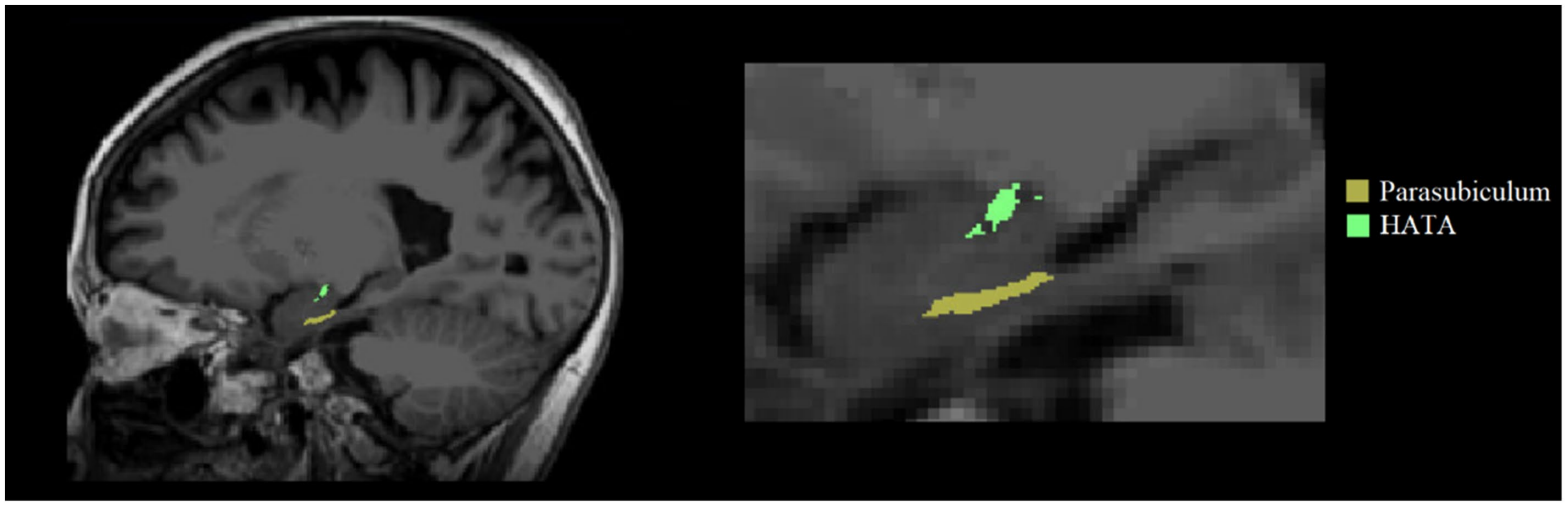
Left two hippocampal subregions with significant volume changes after intervention.

**Figure 3:**
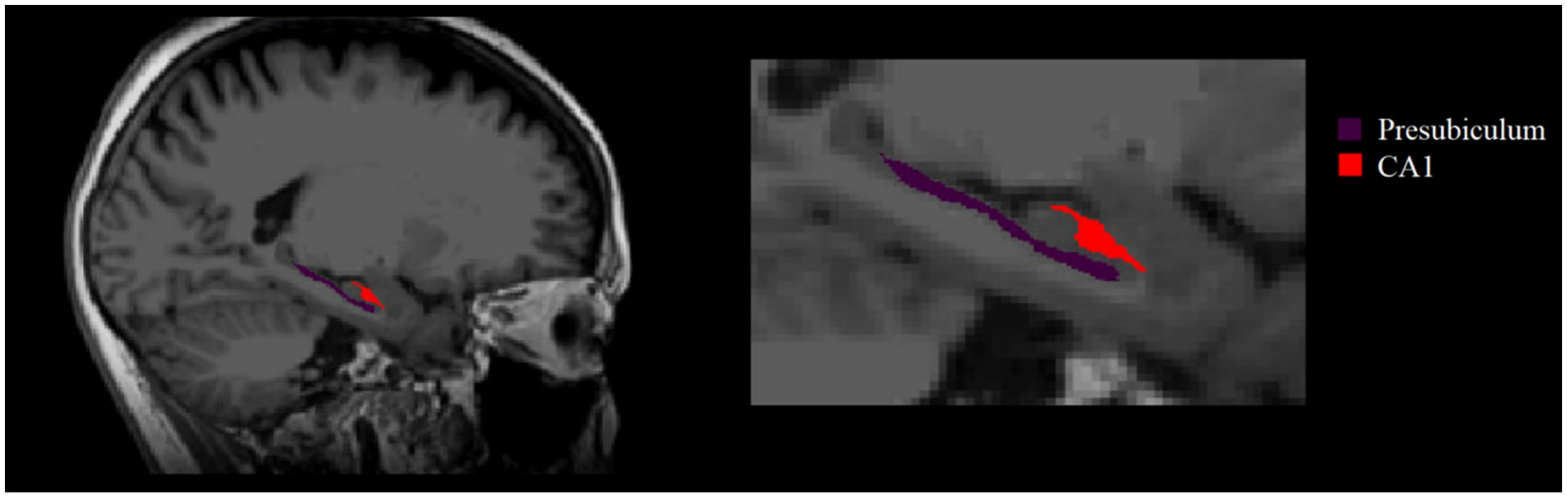
Right two hippocampal subregions with significant volume changes after intervention.

**Figure 4:**
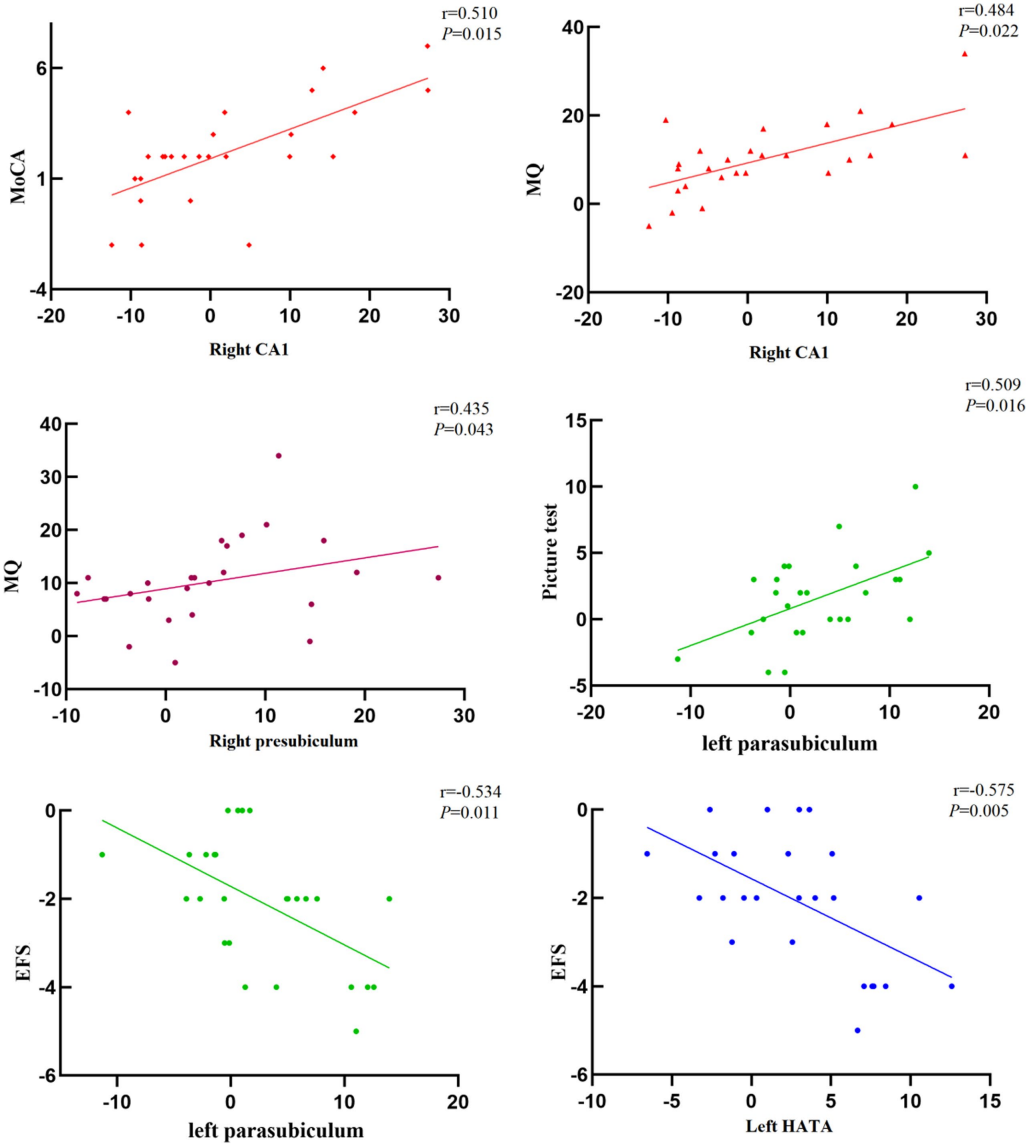
Correlation between the volume change of hippocampal subregion and cognitive frailty in Baduanjin group (*n* = 26).

After 24 weeks of intervention, the change in hippocampal subregion volume before and after intervention was compared between the two groups. The volumes of the left parasubiculum, HATA, right CA1 and presubiculum in the Baduanjin group were significantly increased after intervention, and the volume changes were significantly higher than those in the control group after Bonferroni correction (*p* < 0.05/12; Table 4; Figures 2,3). After adjusting for the influence of sex, age, marital status and years of education, a correlation analysis between the changes in the volumes of the above 4 hippocampal subregions and the changes in cognitive frailty showed that the change in the volume of the right CA1 region in the Baduanjin training group was positively correlated with the change in the MoCA score (*r* = 0.510, *p* = 0.015); the change in the volume of the left parasubiculum and HATA was negatively correlated with the change in the EFS score (*r* = −0.534, *p* = 0.011; *r* = −0.575, *p* = 0.005); the change in the volume of the right CA1 and right presubiculum was positively correlated with the change in the MQ score (*r* = 0.484, *p* = 0.022; *r* = 0.435, *p* = 0.043); and change in left parasubiculum volume was positively correlated with change in the picture subtest scores (*r* = 0.509, *p* = 0.016; Table 5; Figure 4).

**Table 4:** Comparison of the volume change between the two groups (unit: mm^3^).

	BDJ (*n* = 26)	CON (*n* = 24)	*t*	*P*
Hippocampal tail (left)	1.57 ± 30.55	−2.21 ± 21.29	0.503	0.617
Subiculum (left)	5.05 ± 11.61	−5.35 ± 11.69	0.093	0.926
CA1 (left)	−7.31 ± 30.27	−9.71 ± 19.94	0.328	0.744
Hippocampal fissure (left)	1.01 ± 10.80	−3.45 ± 13.07	1.319	0.193
Presubiculum (left)	−3.89 ± 16.92	−5.64 ± 13.87	0.398	0.692
Parasubiculum (left)	2.72 ± 6.06	−2.60 ± 5.37	3.275	**0.002**
Molecular layer of the HP (left)	−6.08 ± 20.75	−8.17 ± 14.15	0.413	0.682
GC-ML-DG (left)	−2.09 ± 11.89	−3.73 ± 10.58	0.512	0.611
CA3 (left)	−1.39 ± 9.62	−1.39 ± 10.02	0.002	0.999
CA4 (left)	−1.20 ± 9.57	−2.60 ± 9.91	0.505	0.616
Hippocampal fimbria (left)	−2.39 ± 9.74	−0.78 ± 8.29	−0.624	0.536
HATA (left)	2.90 ± 4.62	−0.57 ± 3.45	2.996	**0.004**
Total hippocampus (left)	−21.19 ± 94.41	−42.14 ± 71.72	0.878	0.384
Hippocampal tail (right)	0.17 ± 23.25	7.58 ± 19.77	−1.209	0.233
Subiculum (right)	−1.35 ± 10.97	3.73 ± 7.96	−1.860	0.069
CA1 (right)	2.07 ± 11.47	−9.67 ± 13.96	3.261	**0.002**
Hippocampal fissure (right)	1.93 ± 11.15	−3.09 ± 9.35	1.719	0.092
Presubiculum (right)	4.40 ± 8.90	−5.86 ± 13.81	3.149	**0.003**
Parasubiculum (right)	0.98 ± 5.34	0.05 ± 4.71	0.651	0.518
Molecular layer of the HP (right)	−1.36 ± 13.39	−0.34 ± 11.58	−0.287	0.775
GC-ML-DG (right)	−0.69 ± 11.79	0.95 ± 9.56	−0.536	0.595
CA3 (right)	1.17 ± 11.13	0.16 ± 11.83	0.310	0.758
CA4 (right)	−0.60 ± 10.80	1.11 ± 9.12	−0.600	0.551
Hippocampal fimbria (right)	−2.14 ± 7.28	0.80 ± 7.46	−1.409	0.165
HATA (right)	0.32 ± 4.38	−1.09 ± 3.21	1.287	0.204
Total hippocampus (right)	4.91 ± 72.62	3.22 ± 59.17	0.089	0.929

BDJ, Baduanjin exercise group; CON, control group; CA, Cornu Ammonis Subfield; GC-ML-DG, Granule Cell Layer and Molecular Layer of the Dentate Gyrus; HATA, Hippocampal Amygdala Transition Area; Bold type *p* value represented significant.

**Table 5:** Correlation between the volume changes of hippocampal subregion and changes of cognitive ability and physical frailty in Baduanjin group (*n* = 26).

	Left parasubiculum		Left HATA		Right CA1		Right presubiculum	
	*r*	*P*	*r*	*P*	*r*	*P*	*r*	*P*
MoCA	0.032	0.888	0.068	0.764	0.510	**0.015**	0.347	0.113
MQ	0.084	0.709	0.203	0.366	0.484	**0.022**	0.435	**0.043**
Pictures	0.509	**0.016**	0.266	0.232	−0.145	0.520	0.255	0.253
Recognition	−0.029	0.897	0.221	0.323	0.419	0.053	0.157	0.485
Association	−0.115	0.611	−0.123	0.585	0.285	0.198	0.123	0.584
EFS	−0.534	**0.011**	−0.575	**0.005**	−0.151	0.501	−0.323	0.142

Gender, age, marital status and years of education were used as covariates for partial correlation analysis. Bold type *P* value represented significant. MoCA, Montreal Cognitive Assessment; MQ, Memory Quotient; EFS, Edmonton Frailty Scale. Bold type *P* value represented significant.

**Figure 5:**
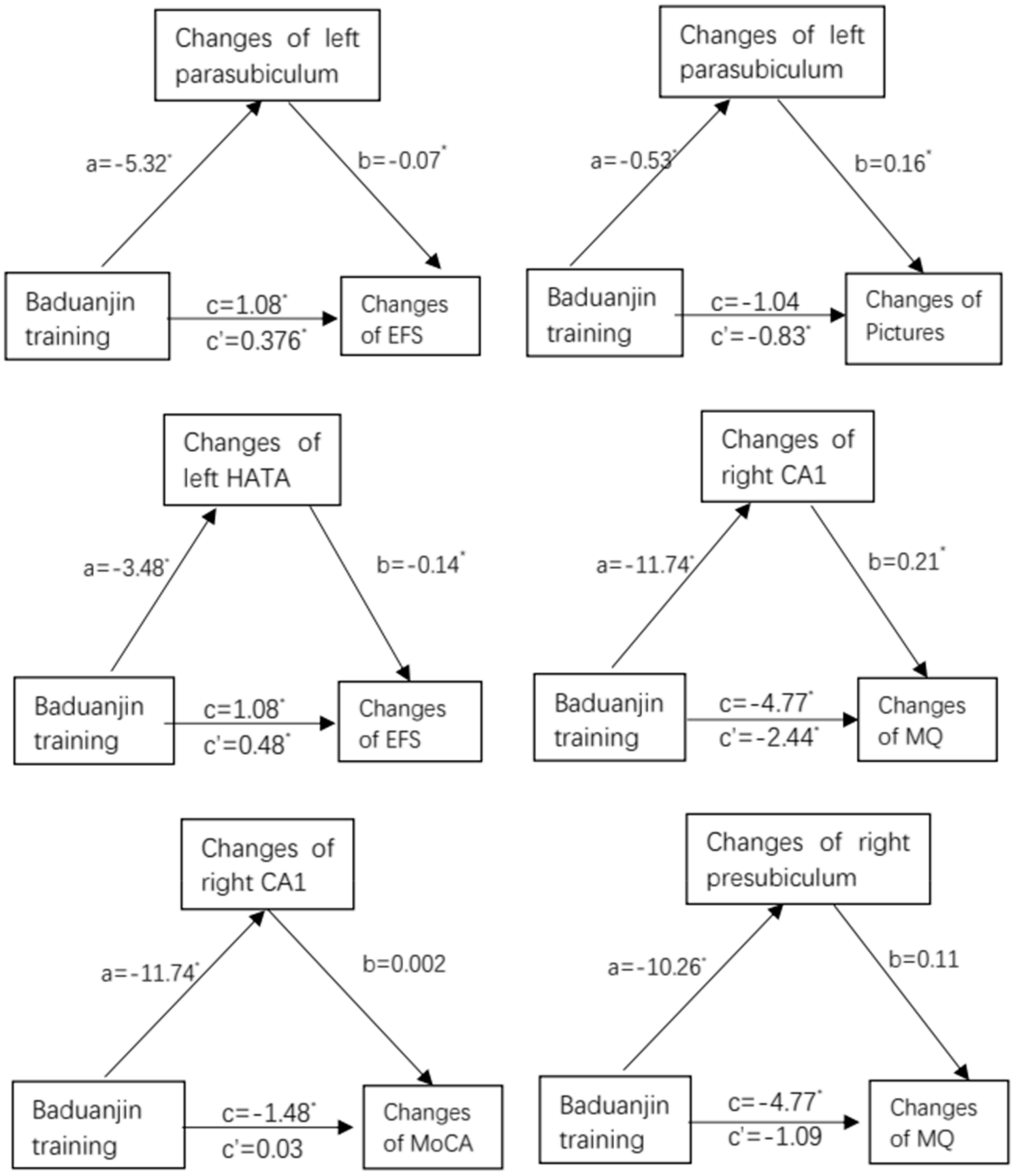
Mediation analysis: path diagram depicting mediation model 4, testing whether changes in changes in hippocampal subregion volume (left parasubiculum, left HATA, right CA1, or right presubiculum) mediate the effect of Baduanjin training intervention vs. control (no specific exercise intervention) on improvement in cognition (MoCA, MQ, or pictures) or physical frailty (EFS). MoCA, Montreal Cognitive Assessment; MQ, Memory Quotient; EFS, Edmonton Frailty Scale; CA1, Cornu Ammonis Subfield 1; HATA, Hippocampal Amygdala Transition Area. a, b, c’ and c are unstandardized path coefficients, and * < 0.05. a-path represent effect of Baduanjin training over hippocampal subregions (left parasubiculum, left HATA, right CA1, or right presubiculum); b-path represent effect of hippocampal subregions over cognition (MoCA, MQ, or pictures) or physical frailty (EFS); c′-path represent indirect effect of Baduanjin training over cognition (MoCA, MQ, or pictures) or physical frailty (EFS); and c-path represent total effect of Baduanjin training over cognition (MoCA, MQ, or pictures) or physical frailty (EFS).

To investigate hippocampal subregions as a potential mediator of Baduanjin exercise training improving cognitive frailty, we performed a mediation analysis using the significant volume changes of hippocampal subregions (i.e., left parasubiculum, left HATA, right CA1, or right presubiculum) as a mediator between groups and the significant scores changes of cognitive or physical frailty measures (MoCA, MQ, or pictures). The analysis results showed the volume changes in left parasubiculum and left HATA mediated the Baduanjin exercise training-induced decrease in the EFS scores with a significant indirect effect (*β* = 0.376, 95% CI 0.024 ~ 0.947; *β* = 0.484, 95% CI 0.091 ~ 0.995); the changes of left parasubiculum and right CA1 mediated the Baduanjin exercise training-induced increase in the picture and MO scores with the significant indirect effects (*β* = −0.83, 95% CI-1.95 ~ −0.002; *β* = −2.44, 95% CI-5.99 ~ −0.32; Figure 5).

## Discussion

This study investigated the changes in hippocampal subregion volumes and cognitive frailty after a 24-week Baduanjin exercise intervention. After 24 weeks of regular Baduanjin intervention, both analysis models (the PP and ITT analysis) showed that the MoCA, MQ, and EFS scores of participants in the Baduanjin exercise training group were significantly improved compared to those in the control group, with a medium to large effect size and significant group by time interaction effect. The MRI data showed that the volume reduction of four hippocampal subregions including the left parasubiculum, left HATA, right CA1 and right presubiculum in the Baduanjin training group was significantly lower than that in the control group. Furthermore, in the Baduanjin training group, the volume change in the left parasubiculum was positively correlated with the change in MQ score and negatively correlated with the change in the EFS index; the volume change in the left HATA was negatively correlated with the change in the EFS index; the volume change in the right CA1 was positively correlated with the change in the MoCA and MQ score; and the volume change in the right presubiculum was positively correlated with the change in MQ score. The mediation analysis showed the volume changes in left parasubiculum and left HATA significantly mediated the Baduanjin exercise training-induced decrease in the EFS scores; the changes of left parasubiculum and right CA1 significantly mediated the Baduanjin exercise training-induced increase in the picture and MO scores. The findings in this study suggest that regular Baduanjin intervention might improve the cognitive ability and physical frailty of community-dwelling older adults with cognitive frailty by modulating the plasticity of some hippocampal subregions.

Baduanjin is a mind–body exercise based on traditional Chinese medicine. A systematic review showed that Baduanjin exercise was safe and effective in enhancing the overall cognitive function and memory of middle-aged and older adults (Wang et al., 2021). One study also found that a 6-month Baduanjin training intervention had a positive effect on increasing brain gray matter in the temporal, frontal, parietal, medial occipital, cingulate and angular gyrus, and improving cognitive function in older adults with MCI. Furthermore, the increase in the right medial temporal gyrus was significantly correlated with the improvement in cognitive function (Zheng et al., 2021). Another study reported that Baduanjin training could change the functional connection of the dorsal attention network in patients with MCI and improve their attention (Xia et al., 2019). Current findings showed that the 24-week Baduanjin exercise intervention significantly improved global cognitive ability, memory, and physical frailty in community-dwelling older adults with cognitive frailty and significantly reduced the atrophy of some hippocampal subregions. These findings further support previous studies and suggest a potential mechanism by which Baduanjin exercise improves cognitive frailty.

The CA1 subregion of the hippocampus functions in the separation of spatial coding and recall processes by processing spatial and temporal memory at the same time as an independent neural network in the process of memory coding (Eichenbaum, 2017; Tanaka et al., 2018) and plays a special role in encoding episodic memory information (Schlichting et al., 2014). Existing studies have shown that exercise can affect the structural plasticity of the CA1 region and improve cognitive function (Woodward et al., 2018). The present study also found that 24 weeks of Baduanjin exercise intervention can improve the cognitive and physical function of older adults with cognitive frailty and reverse the decrease in CA1 volume. Other studies suggest that running may induce hippocampal neurogenesis and synaptic plasticity by increasing the complexity and number of dendritic spines in the dentate gyrus, CA1 and entorhinal cortex (Opendak and Gould, 2015). A systematic review revealed that mind–body exercise modulated brain structure and functional connectivity mainly in the hippocampus/medial temporal lobe, as well as the cognitive control and default mode networks, which might underlie the beneficial effects of such exercises on the health of individuals (Zhang et al., 2021).

The presubiculum of the hippocampus is a part of the subiculum, which is the transition region from the parahippocampus to the hippocampus. Studies have shown that smaller hippocampal and presubiculum volumes could predict the conversion of Parkinson’s patients to dementia, while the hippocampal, presubiculum and parasubiculum volumes of Parkinson’s patients with dementia were significantly decreased, and the decrease in these volumes was positively correlated with the decrease in cognitive ability (Low et al., 2019). Another study also found that from the early stage of Alzheimer’s disease, the subiculum and presubiculum of the hippocampus have atrophied (Carlesimo et al., 2015). Therefore, structural changes in the presubiculum might play an important role in predicting cognitive impairment. Our study showed that Baduanjin could slow the volume decrease of the right presubiculum, and there was a positive correlation between the right presubiculum and MQ score (*r* = 0.435). The volume of the presubiculum is positively correlated with the ability of observation, which could also prevent the decline of memory and enhance its persistence (Barry et al., 2021).

The parasubiculum is a small, narrow structure located between the presubiculum and medial entorhinal cortex, which is responsible for spatial navigation and memory (Sammons et al., 2019). Atrophy of the parasubiculum was only observed in older adults with Alzheimer’s disease; thus, researchers believe that a significant decrease in the volume of the parasubiculum may be a potential biomarker of dementia (Zheng et al., 2018). Additionally, atrophy of the parasubiculum may affect the integrity of the hippocampal-amygdala network, which is the basis of information processing; therefore, it may play a key role in cognitive processes (Foo et al., 2017). Animal experiments also found that the parasubiculum may be more involved in real-time spatial information processing than in long-term information storage (Tang et al., 2016). The results of the present study showed that there was a positive correlation between short-term memory test results and parasubiculum volume (*r* = 0.509). This suggests that the integrity of the parasubiculum structure may be closely related to the function of immediate spatial information processing.

Hippocampal amygdala transition area anatomically connects the hippocampus to the amygdala and has a regulatory effect on muscle atrophy. It has been reported that the HATA of amyotrophic lateral sclerosis (ALS) patients with central amyotrophic and myasthenic lesions presents significant atrophy (Christidi et al., 2019). The hippocampal input to the amygdala originates from the CA and mainly projects to the amygdala area, the accessory nucleus of the amygdala and the ventral basal nucleus through the HATA and contains immature neuronal populations, which may be the cellular mechanism behind the plasticity of context learning and emotional memory (Fudge et al., 2012). Our results showed that 24 weeks of Baduanjin exercise intervention may reduce physical frailty and increase the HATA volume. Furthermore, there was a significant negative correlation between HATA and EFS score (*r* = −0.575), which means that a smaller HATA volume indicates a frailer individual. Our findings suggest that the mechanism by which Baduanjin improves the physical frailty of older adults with cognitive frailty might be associated with an increase in the HATA volume.

In terms of other hippocampal subregions. CA3 and CA4 play the important role in the encoding of new spatial information with short-term memory (Kesner, 2007). The GC-ML-DG mainly receives cortical input from the entorhinal cortex and projects to CA3 pyramidal cells, also is a key brain region associated with the stress response, pathology of depression, and antidepressant response (Tuncdemir et al., 2019). The subiculum has functional properties seemingly independent from the rest of the hippocampus, and has a substantial contribution to interregional communication and behavioral performance (Matsumoto et al., 2019). Hippocampal fimbria, as a structural bridge between the hippocampus and other brain regions, is key to hippocampus preserving memory (Chauhan et al., 2021). Hippocampal fissure, also called the hippocampal sulcus, is a kind of narrow furrow to separate the gyri of brain (Humphrey, 1967); and its width may negatively related to the atrophy of the hippocampal subfields (Li et al., 2018). Growing evidence indicates that physical exercise could profoundly increase hippocampal neurogenesis, by altering neurochemistry and function of newly generated neurons (Pereira et al., 2007; Lafenetre et al., 2011; Farioli-Vecchioli and Tirone, 2015; Ma et al., 2017; Firth et al., 2018). In our present trial, we did not find a 24-week Baduanjin exercise training had a significant effect in improving structures of those hippocampal subregions including CA3, CA4, Subiculum, hippocampal fissure, and, hippocampal fimbria. A recent study reported that the exercise type is an important factor affecting the effects of exercise on the hippocampus (Tsuchida et al., 2022). Another possible reason results from the small samples of our current trial. Therefore future studies with a larger samples is need to further determine the relation of Baduanjin exercise intervention with those hippocampal subregions.

## Limitations

Some limitations of this study should be recognized. First, we used hippocampal subregion analysis to explore the possible mechanism by which Baduanjin improves cognitive frailty. Instead of analyzing the problem from the perspective of the whole brain, it only focuses on the hippocampus. Second, because some older adults who participated in the study did not correctly report their metal implantation conditions at enrollment or were unwilling to perform the MRI scan at baseline assessment, leading to a reduction in the MRI sample size. We compared the baseline characteristics between two groups among three data sets, in which contained all 102 participants, 73 participants who were willing to do MRI scan, and 50 participants who completed two MRI scan, respectively, and the results showed little variability among three data sets and good balance between two groups for each data set. In addition, we also analyzed effect of Baduanjin exercise intervention on cognitive ability and physical frailty outcomes using two analysis models, and the findings were equivalent. Even so, the attrition bias was possible unavoidable due to unknow confounders. In future research, the sample size should be expanded to improve the stability of the results. Third, this study only focuses on the hippocampal subregions and those hippocampal subregions that showed no significant changes were not discussed. Future studies are needed to further explore more brain structures. Fourth, considering the difficult of recruitment and study to be conducted, this study did not use another experimental control group with another exercise type to determine the effect of Baduanjin different from other exercise type. Finally, EFS is a frailty assessment tool (including cognition, social support, mood indicators other than physical indicators), therefore it has a limitation on accurately assessing physical frailty characterized by the vulnerability of strength, endurance, and physiological functions. Therefore, those findings of this trial should be explained cautiously. Future study should compare Baduanjin training with other exercise type to identify its exclusive effect. In addition, participants in the control group of this study did not to receive any specific exercise intervention (maintain their original life habit), which would result in ethical implications.

## Conclusion

The 24-week Baduanjin exercise intervention effectively improved the cognitive ability and reduced the physical frailty of community-dwelling older adults with cognitive frailty and slowed the atrophy of hippocampal subregions, including the left parasubiculum, left HATA, right CA1 and right presubiculum. The mechanism by which Baduanjin improves cognitive frailty of older adults may be associated with changes in the structural plasticity of hippocampal subregions. This results also provide potential theoretical support for the application of Baduanjin exercise to intervene the cognitive frailty of community-dwelling older adults.

## Data availability statement

The original contributions presented in the study are included in the article/Supplementary material, further inquiries can be directed to the corresponding author.

## Ethics statement

The studies involving human participants were reviewed and approved by Second People's Hospital Affiliated to Fujian University of Traditional Chinese Medicine. The patients/participants provided their written informed consent to participate in this study.

## Author contributions

GZ designed this study and responsible for coordinating and monitoring the process. MW and RX wrote the manuscript. HL, YY, and PQ manage maintenance and data analysis. All authors contributed to the article and approved the submitted version.

## Funding

This study was supported by the National Natural Science Foundation of China (http://www.nsfc.gov.cn, No. 82074510) and GuangDong Basic and Applied Basic Research Foundation (2021A1515110764).

## Conflict of interest

The authors declare that the research was conducted in the absence of any commercial or financial relationships that could be construed as a potential conflict of interest.

## Publisher’s note

All claims expressed in this article are solely those of the authors and do not necessarily represent those of their affiliated organizations, or those of the publisher, the editors and the reviewers. Any product that may be evaluated in this article, or claim that may be made by its manufacturer, is not guaranteed or endorsed by the publisher.

## Footnotes

^1^ http://www.chictr.org.cn/index.aspx

^2^ http://www.freesurfer.net/swiki/HippocampalSubfieldsAndNucleiOfAmygdala
