# Supplementary material for: Baduanjin exercise modulates the hippocampal subregion structure in community-dwelling older adults with cognitive frailty
Source: Front Aging Neurosci. 2022 Dec 19;14:956273. doi: 10.3389/fnagi.2022.956273 (PMC9806122; doi:10.3389/fnagi.2022.956273)
Supplement: Supplementary file 1 [file Table_1.docx]

| Supplement Table 1 Comparison of baseline characteristics between two groups among three data sets (ratio/**$\overline{X}\pm S$**/*M*(*P*_25_~*P*_75_)) | | | | | | | | | |
| --- | --- | --- | --- | --- | --- | --- | --- | --- | --- |
| Characteristics | All participants | | | Participants who were willing to do MRI scan | | | Participants who competed two MRI scan | | |
|  | BDJ(n=51) | CON(n=51) | *P* | BDJ(n=37) | CON(n=36) | *P* | BDJ(n=26) | CON(n=24) | *P* |
| Age (year) | 67.68±5.19 | 65.35±5.15 | **0.021** | 67.00±5.25 | 65.75±5.20 | 0.310 | 67.31±5.58 | 64.71±5.07 | 0.065 |
| Gender (male/ female) (n) | 19/32 | 20/31 | 0.839 | 13/24 | 13/23 | 0.931 | 9/17 | 12/12 | 0.271 |
| marital status (married / widowed) (n) | 49/2 | 51/0 | 0.987 | 36/1 | 36/0 | 0.321 | 25/1 | 24/0 | 1.00 |
| BMI (kg/m^2^) | 23.75±2.33 | 24.32±3.22 | 0.310 | 23.52±2.21 | 24.27±3.24 | 0.251 | 23.47±2.28 | 24.45±3.28 | 0.225 |
| Average years of education (year) | 10.90±2.81 | 10.02±2.90 | 0.124 | 11.05±2.92 | 10.17±2.89 | 0.196 | 11.58±2.79 | 10.25±2.92 | 0.108 |
| Beck Depression Scale Index* | 3(2~5) | 3(3~5) | 0.908 | 4(2~5) | 4(3~5) | 0.758 | 3.5(1.75~5) | 3.5(3~5) | 0.670 |
| Global deterioration scale（II/III）(n) | 34/17 | 36/15 | 0.670 | 24/13 | 26/10 | 0.499 | 17/9 | 19/5 | 0.278 |
| BDJ, Baduanjin exercise group; CON, control group. BMI, Body Mass Index.* Data were performed by Mann–Whitney U tests. | | | | | | | | | |
